# Novel mutatıons and diverse clinical phenotypes in recombınase-activating gene 1 deficiency

**DOI:** 10.1186/1824-7288-38-8

**Published:** 2012-03-16

**Authors:** Necil Kutukculer, Nesrin Gulez, Neslihan Edeer Karaca, Guzide Aksu, Afig Berdeli

**Affiliations:** 1 Department of Pediatrics, Faculty of Medicine, Ege University, 35100 Bornova, Izmir, Turkey

**Keywords:** Recombinase-activating genes, Immunodeficiency, BCG

## Abstract

**Background:**

Severe combined immunodeficiency is within a heterogeneous group of inherited defects throughout the development of T- and/or B-lymphocytes. Mutations in recombinase-activating genes 1 or 2 (RAG1/2) represent approximately 10% of all SCID cases. RAG1/2 are essential for V(D)J rearrangement of the B- and T-cell receptors.

**Objectives:**

The aim of this study was to review clinical, immunological and molecular findings of Turkish SCID patients with RAG1 defects and to draw attention to novel mutations, genotype-phenotype correlations and the high rate of BCG infections within this group.

**Methods:**

Eleven patients (F/M: 6/5) were included. Molecular, immunological and clinical data were evaluated.

**Results:**

Five patients were classified as T-B-NK + SCID, four patients as T + B-NK + SCID (two of these patients were diagnosed as classical Omenn syndrome) and two patients as T + B + NK + SCID with respect to clinical presentations and immunological data. Mean age of the whole study group, mean age at onset of symptoms and mean age at diagnosis were: 33.0 ± 42.8, 3.1 ± 3.3 and 10.4 ± 13.5 months, respectively. Consanguinity rate was 54%. Some novel mutations were found in RAG1 gene in addition to previously reported mutations. Genotype-phenotype correlation was not significantly apparent in most of the cases. BCG infection was observed in 36.4% of patients (two BCG-osis and two BCG-itis).

**Conclusion:**

Epigenetic factors such as compound genetic defects, enviromental factors, and exposure to recurrent infections may modify phenotypical characteristics of RAG deficiencies. Inoculation of live vaccines such as BCG should be postponed until primary immunodeficiency disease is excluded with appropriate screening tests in suspected cases.

## Background

Severe combined immunodeficiency (SCID) syndromes embrace common phenotypic presentation of a range of genetic disorders [1]. According to recent immunological and genetic findings, SCID can be subdivided into 11 conditions. These conditions are abnormally increased apoptosis of the lymphocytes (reticular dysgenesis caused by adenylate kinase 2 (AK2), adenosine-deaminase-deficiency), defects of cytokine signaling (X-linked SCID, IL7-receptor-α, JAK3- deficiency), defects in T-cell-receptor (TCR) assembly and signaling (RAG1/2, DNAPKcs, Artemis and Cernunnos, CD3 defects) and general T-cell signaling defects associated with calcium release-activated Ca++ channels (CRAC) and yet unclassified defects such as the deficiency of RNA component of mitochondrial RNA processing endoribonuclease (RMPR) also known as Cartilage-Hair-Hypoplasia [2-4].

Mutations in recombinase activating genes 1 or 2 (RAG1/2) represent approximately 10% of all SCID cases [5]. These genes are located within human11p13 chromosome. Their products; RAG1 and RAG2 proteins are essential for V(D)J rearrangement of the B (BCR) and TCR during T and B cell development [6-8].

The first mutation in the RAG1 gene was identified in 1996 by Schwarz et al [9]. Complete RAG deficiency (RAGD) with no V(D)J (< 1% recombination activity of wild type) is associated with classical SCID and absence of T and B cells. It is estimated that RAG1/2 is involved in approximately 50% of all patients with T-B-NK + SCID phenotype [10]. In RAGD with residual V(D)J activity (> 1% recombination activity of wild type), several clinical and immunological subtypes have been described; such as RAGD with skin inflammation and αβ T-cell expansion (classical Omenn syndrome), RAGD with skin inflammation and without T-cell expansion (incomplete Omenn syndrome), RAGD with γδ T-cell expansion and RAGD with granulomas [2,3,11]. B cell levels and immunoglobulin concentrations are almost normal or only slightly reduced in patients with hypomorphic mutations [12]. In recent studies, autoimmune cytopenia and resistant CMV infection are reported in hypomorphic RAG mutated patients [12-14]. These patients usually present with severe and life-threatening bacterial, viral and fungal infections in early infancy regardless of SCID type and the underlying genetic defect. Some patients with RAG deficiency may present later in life as combined immunodeficiency with granuloma formation [15]. Patients with SCID also experience opportunistic infections of mycobacterium species and complications after vaccination with Bacille Calmette-Guerin (BCG).

The aim of this study was to review clinical, immunological and molecular findings of Turkish SCID patients with RAG1 defects admitted to a Pediatric Immunology Department in the western region of the country and to draw attention to novel mutations, genotype-phenotype correlations and high frequency of BCG infections.

## Patients and methods

### Patients

Eleven patients with the diagnosis of SCID in respect to severe upper and lower respiratory tract infections from 2002 to 2010 in Ege University Faculty of Medicine, Department of Pediatric Immunology, Izmir, Turkey; were reviewed in this study. An evaluation sheet was used to summarize demographic information of patients including name, gender, date of birth, age of onset of symptoms, clinical symptoms, age at diagnosis, family history and consanguinity, previous history of medications and vaccination, and laboratory and molecular data. The patients were diagnosed and classified according to clinical and laboratory criteria of SCID reported by IUIS Expert Committee on Primary Immunodeficiencies [16].

All patients were screened for mutations in *RAG1, RAG2 *gene. Ten age-matched, healthy people (mean age: 27.4 ± 2.1 years) served as controls for the analyses of genetic data. This study was approved by the Ethics Commitee at Ege University, and an informed consent was obtained for each participating patient.

### Cellular and immunological assays

Complete blood count with peripheral blood smear evaluation, serum immunoglobulins by nephelometry (Dade Behring BNII Nephelometer Analyzer, Germany), antibody response to previous vaccines, and lymphocyte phenotyping (T, B, and NK cells) by flow cytometry (FACSCalibur, Becton-Dickinson, USA) were the standard laboratory investigations performed for our patients.

### Clinical samples and DNA isolation

Peripheral blood samples were obtained from the patients by venipuncture. Genomic DNA was then extracted from the blood samples using standard techniques [17].

### Mutations analyses at the RAG-1 and RAG-2 loci

Because of the restricted expression of *RAG-1 *and *RAG-2 *genes and the fact that the coding region of each gene is contained in a single exon, coding sequences were amplified from genomic DNA. Primers were designed for the amplification of the *RAG *genes based on the sequences reported in databases (*RAG-1*, M29474; *RAG-2*, M94633). *RAG *gene sequence information was obtained as previously specified [18]. Alternatively, the *RAG-1 *gene was amplified in 2 segments (94-1852 and 1781-3262), and the *RAG-2 *gene was amplified in one segment (1201-2922) with the following primers: *RAG-1*-90 F, CTG AGC AAG GTA CCT CAG C; *RAG-1*-1852R, GCC TTC CAA GAT GTC TTCTTC; *RAG-1*-1781 F, GCAAAG AGG TTC CGC TAT GA; *RAG-1*-3262R, CAT AAG TGG TTG CCC TAC TT; *RAG-2*-1201 F, ATG TCT CTG CAG ATG GTA AC; *RAG-2*-2922R, CTG GCC CTT AAT TCA TGT AAC. Sequencing was performed directly on the PCR products purified from the gel with the Thermosequenase kit (Amersham Pharmacia Biotech UK, Buckinghamshire, United Kingdom). For patients who were compound heterozygotes, mutations were confirmed either by restriction analysis or by analysis of several clones from PCR amplification products cloned in TA vector (Invitrogen) and sequenced by the dideoxynucleotide chain termination method using the Sequenase kit (USB), as previously described [18].

SPSS was used for statistical analyses (Windows Version 15.0, SPSS Inc., Chicago, IL).

## Results

Eleven patients (F/M: 6/5) with SCID or Omenn Syndrome who had RAG mutations were analyzed. Their demographic findings are listed in Table 1. Among them, two have been previously reported [14]. Five patients were classified as T-B-NK + SCID, four patients as T + B-NK + SCID (two of these patients were diagnosed as classical Omenn syndrome) and two patients as T + B + NK + SCID with respect to clinical presentations (Table 1) and immunological data (Table 2). Mean age of the whole study group, mean age at onset of symptoms and mean age at diagnosis were; 33.0 ± 42.8, 3.1 ± 3.3 and 10.4 ± 13.5 months, respectively. Consanguinity was present in 6 (54%) of 11 patients and all of them were second degree relatives. Positive family history for immunodeficiency was determined in five patients (45%).

**Table 1:** Demographic and clinical findings of patients with RAG1 defects

SCID type	n	Gender (f/m)	Mean Age months	Mean age onset of symptom months	Mean age at diagnosis	consanguinity (n)	Positive autoimmunity (n)	CMV infect (n)	BCG infect (n)	BMT (n)	Follow up		
											
											Ex (n)	IVIG (n)	Healthy (n)
**T-B-NK+**	5	3/2	10,4 ± 9,4	2,6 ± 1,7	5,9 ± 4,7	3	1	0	1	3	4	1	0

**T+B-NK+**	Omenn 2	1/1	8,5 ± 9,1	0,6 ± 0,5	1,5 ± 1,4	0	1	0	1	0	2	0	0
	
	Other phenotype. 2	1/1	98 ± 72,1	7.0 ± 7.0	33 ± 21,2	1	0	0	1	0	0	2	0

**T+B+ NK+**	2	1/1	49 ± 4,2	2,7 ± 0,3	8.0 ± 2,8	2	1	2	1	2	0	1	1

**Total**	11	6/5	33 ± 42,8	3,1 ± 3,3	10,4 ± 13,5	6	3	2	4	5	6	4	1

**Table 2:** Immunological and genetic findings of patients with RAG1 defects

	T-B-NK+ (n = 5)					T + B-NK+ (n = 4)				T + B + NK+ (n = 2)	
								
						Other phenotype n(2)		Omenn n(2)			
	
	Patient 1	Patient 2	Patient 3	Patient 4	Patient 5	Patient 6	Patient 7	Patient 8	Patient 9	Patient 10	Patient 11
**IgG (mg/dl)**	371	546	546	517	312	1372	1840	1080	662	159	1590

**IgA (mg/dl)**	< 6	< 6	< 6	< 6	< 6	22	219	49	< 6	8	52

**IgM (mg/dl)**	< 17	< 17	< 17	< 17	< 17	222	120	179	< 17	52	75

**WBC**	7800	8220	532	8280	6780	5900	8740	24700	19200	15500	11000

**Abs.lymp h./mm^3^**	1028	657	180	3120	650	3835	1748	9386	6144	7630	5500

**CD3+ %**	10	0	0	0	0	56	78	92	78	59	51

**CD3 + CD 4+ %**	0	0	0	0	0	11	17	19	33	37	9

**CD3 + CD 8+ %**	10	0	0	0	0	50	55	60	47	19	39

**CD19+ %**	0	0	0	0	0	0	1	0	0	30	36

**NK + % CD16 + 56+)**	75	43	90	78	63	43	12	2	11	7	16

**HLA DR + T cell %**	10	58	0	0	0	45	67	85	73	12	37

**Molecular analyses**	p.R394Q/p.R394Q homozy. mutation	p.R394Q/p.R394Q homozy. mutation	p.R776Q, 3047-3049 del GCC	p.H249R (homozy) mutation? Polymorphism?	p.R394Q/p.R394Q homozy. mutation	p.H249R/p.K820R (compound heterozy.) mutation? Polymorphism?	p.H249R/p.K820R (compound heterozy) mutation? Polymorphism?	p.H249R (heterozy) mutation? Disease causing? Polymorphism?	p.Q248X/p.Q248X homozy. mutation	p.H249R (heterozy) mutation? Disease causing? Polymorphism?	P85fs32 × = del A256/A257

The most common symptoms were pneumonia (n = 10), chronic diarrhea (n = 6), infections plus dermatitis (n = 3), infections plus moniliasis (n = 3) or all of these (n = 3). The respiratory tract diseases were the most frequent infection type. Lymphadenopathy and hepatosplenomegaly were observed in 36% and 90% of RAGD patients, respectively. Auotoimmunity was recorded in three patients. CMV infection was detected in two patients with T + B + NK + phenotype.

Absolute lymphocyte counts and lymphocyte subset distributions in the RAGD patients are shown in Table 2. Lymphopenia was documented in 36% of all patients (in 80% of T-B-NK + SCID patients, and 25% of T + B-NK + SCID patients). A high variability in the total lymphocyte count was documented in these groups. Data on the expression of activation markers (HLA-DR) was obtained for the subgroups of RAGD SCID patients. In particular, the T + B-NK + group showed increased percentages of HLA-DR + cells (100%). Two groups (T + B- and T-B+) of patients with RAG1 defects had very low proportions of CD19 cells. The highest NK cell percentages were found in patients with T-B- SCID (73.2%). Eosinophilia, a well-known feature of Omenn Syndrome, was observed in 2/4 of the T + B-patients group, in 1/5 of T-B- patients and 1/2 of T + B + patients. None of the patients have been shown to produce specific antibodies against vaccines, such as tetanus toxoid, *Haemophilus Influenza type B *or *hepatitis B*.

### Genotype-phenotype correlation

We investigated the mutations in RAG1 and RAG 2 genes in SCID patients and the control group (Table 2) and compared them with the clinical phenotypes. The following findings were detected in 11 SCID patients:

#### Two novel mutations in four patients

Homozygous mutation of *p.R394Q/p.R394Q *was found in three patients of the T-B- group (patient 1- 2-5). Two of these patients and their parents (no. 1 and 5) were cousins. Lymphopenia, hypogammaglobulinemia, early onset of disease symptoms were significant in these three patients.

Another mutation which was not reported before, in T-B- SCID group, was *p.R776Q, 3047- 3049 del GCC *(patient 3). This female patient had chronic diarrhea beginning from the neonatal period. The other clinical findings were hepatomegaly, splenomegaly, dermatitis, anal fissure and failure to thrive. Direct Coombs test was positive. In addition to chronic pulmonary infection, lymphadenopathy was observed both in computerized tomography of the thorax and the axillary area. *Mycobacterium bovinum *was detected in her sputum and she began to receive antimycobacterial therapy after the diagnosis of BCG infection.

#### Two previously reported mutations in two patients

Homozygous mutation of *Q248X *in our Omenn Syndrome patient (case no.9) has been reported before in T-B-NK + SCID and Omenn Syndrome cases. Similar to the previously reported cases, *P85fs32X (del A256/A257) *mutation was found in one (case no.11) of our leaky SCID patients. This case had refractory CMV infection, autoimmunity and elevated gamma-delta T cells.

#### Amino acid changes: Mutations or polymorphisms? (Five patients)

*p.H249R *amino acid change (homozygous) was detected in a T-B-NK + patient (no.4), who was admitted on 40/365 day because of pulmonary infection and moniliasis and underwent BMT when he was three months old.

*p.H249R *amino acid change (heterozygous) was observed in two of our cases (patients 8 and 10), one with Omenn Syndrome and one with T + B + SCID. The Omenn Syndrome case had all the classical clinical and laboratory findings. He had early-onset generalized erythroderma, failure to thrive, protracted diarrhea, hepatosplenomegaly, lymphadenopathy, and eosinophilia. This case had developed left axillary lymphadenopathy six weeks after BCG infection. *Mycobacterium bovinum *was isolated from the lymph node (BCGitis). The other case with T + B + SCID had recurrent pulmonary infections, splenomegaly and CMV infection. She received the BCG vaccine before the diagnosis of SCID and developed bilateral axillary lymphadenopathy three months after vaccination. Granulomatous lymphadenitis was observed in lymph node biopsy and *Mycobacterium bovinum *was isolated (BCGitis). Analysis of the parents of these two patients revealed that the mothers also had the same findings.

*p.H249R/p.K820R *amino acid changes (compound heterozygous) were observed in two patients of T + B- group. The male case (patient 7) had diarrhea, recurrent bronchiolitis and autoantibodies. Several nodules were observed on his shoulder and gluteal area when he was three years old. *Mycobacterium Bovinum *was isolated both from biopsy of nodules and from bronchoalveolar lavage fluid (BCGosis). The female case (patient 6) had recurrent pulmonary infections, diarrhea, hepatomegaly, splenomegaly, and bronchiectasis,. She is now 13 years old and receiving regular intravenous immunoglobulin therapy.

Mutations or amino acid changes have not been observed in the healthy control group. There was no mutation in RAG 2 gene in either the patients' or control groups.

### RAG1base and RAG2base mutation databases

All reported *RAG-1 *mutations were recorded into databases called *RAG1*base and *RAG2*base. These databases were constructed according to http://www.uta.fi/imt/bioinfo/RAG1base and/RAG2base.

### Mortality and survival

Five patients underwent bone marrow (BMT) or umblical cord stem cell transplantation. These patients received bone marrow from sibling (n = 1), haploidentical mother (n = 2) (Patient 11 had 2 BMTs), haploidentical father (n = 1) and matched unrelated (n = 2) donors. Mean follow-up duration after transplantation was 9.0 ± 8.9 months. Two patients deceased after transplantation and 4 patients deceased before transplantation during follow-up. Engraftment was not achieved in two cases. One of them was a T + B + NK + leaky SCID male patient and the donor was his mother, and the other patient was a T-B-NK + patient and the donor was his brother. One patient (T + B + NK + leaky SCID female patient) has fully recovered after BMT. Two patients are currently being followed by prophylactic IVIG replacements and they are waiting for the results of matched unrelated donor screening.

## Dıscussıon

Mutations in RAG1 or RAG2 represent approximately 10% of all SCID cases and most of them are T-B-NK + SCID [16]. For our patients, the frequencies of T-B-NK+, T + B-NK + and T + B + NK + SCID phenotype were 45%, 36% and 18%, respectively. All of our patients fullfilled previously defined clinical criteria for SCID. Consanguinity (54%) and family history for immunodeficiency (45%) were very high within our patient group. A high rate of consanguineous marriages (21%) is a social and health problem in Turkey as a result of some traditional concepts, especially in eastern regions. Female/male ratio was found to be 1.2. Because of these findings and the effect of high consanquinity rate, RAGD is previously reported to be inherited in an autosomal recessive pattern [18-20].

To date, at least 55 different hypomorphic RAG mutations have been described; however, clear correlation between the type of mutations and clinical presentations has not been observed. This may be due to specific mutations or other genetic factors, or epigenetic mechanisms. The mutations identified, both in RAG1 and RAG2 genes; can either be severe, leading to null alleles, or mild, leading to hypomorphic alleles that can still maintain a residual enzymatic activity of RAG1/2 proteins. Null mutants typically predominate in classical T-B-SCID, with no productive rearrangement of the T cell receptor (TCR) or B-cell receptor (BCR); while missense mutations predominate in Omenn syndrome and leaky SCID [21,22]. The same mutations in different individuals usually lead to similar phenotypes. There are also a few cases in which the same mutation gives rise to a different clinical presentation. This suggests that yet unknown factors may play a role in determining the clinical picture and outcome [21-25].

The hypomorphic mutation*, P85fs32X (del A256/A257) *(old nomenclature is *k242fsX246 del A368/A369)*, demonstrated in our eleventh patient, was previously reported by Villa A et al [13]. In another report by de Villartay et al, four unrelated patients, with the same hypomorphic RAG1 mutations were presented [26]. The immunological phenotype of Villartay et al's patients consisted of a restricted T cell repertoire, with TCR〈β lymphopenia, but markedly high levels of TCRγ™ T cells. Severe, disseminated CMV infection and autoimmune blood cell manifestations shaped their clinical phenotype. Our patient with the same hypomorphic mutation had almost the same clinical and laboratory findings, showing a clear correlation between genotype and phenotype as these 4 patients.

Sobacchi et al [2] have reported 24 RAGD cases and 16 novel mutations in 2006. One of the mutations in this report was a *p.Q248X *homozygous mutation observed in a T-B-SCID case. Our patient (no.9) with the same mutation had the characteristic findings of Omenn Syndrome. Asai et al have recently reported (2011) 4 missense and 1 nonsense mutations in RAG1 of three SCID children [27]. An atypical SCID case with maternal T cell engrafment carried a homozygous novel *E770K *mutation [27]. One case with atypical Omenn syndrome was compound heterozygous bearing novel *R142X *and *R396H *mutations [27]. The last case with γδ T- CMV infection was a compound heterozygote bearing novel *R474C *and *L732F *mutations in RAG1 [27]. None of our cases was carrying these mutations.

Pico-Knijnenburg et al [28] have reported a family with three siblings who were identified by early onset of infections, pneumonia, sepsis, generalized dermatitis, malnutrition and diarrhea. There was a strong reduction of T and B cells and a complete block in precursor B cell differentiation in bone marrow that showed a V(D)J recombination defect [28]. In all siblings, a heterozygous mutation in RAG1 was identified (c2571A > G; p.Cys328Tyr) [28]. They thought that, in case of heterozygous mutations of RAG1 gene, there might be another defect on the second allele, which was not picked up with routine methods such as PCR and sequencing of the coding region. In her cohort of patients, Villa A also has observed Omenn cases carrying mutations on only one allele and no mutations in the promoter regions or in other genes involved in VDJ recombination (personal communication). According to her, a careful single nucleotide polymorphisms (SNPs) analysis would also be useful to clarify these patients' defects.

*H249R *(one homozygous and two heterozygous patients in our study) and *K820R *(two compound heterozygous patients) variants have been observed in our patient group, but not in our small healthy control group. Notarangelo LD (Boston Children's Hospital, USA) has found several normal subjects with these variants and he thought that they could not be disease-causing-polymorphisms, (personal communication). Our five patients presented with classical signs, symptoms and laboratory findings of SCID and our control group for the molecular analyses included ten people only. Thus, we suggest that a future study disclosing the functional activity of human RAG mutants would tell us if these variants are disease causing or polymorphisms. In addition, analyses of RAG1 gene in at least one-hundred healthy Turkish people will lead us to know more about the disease activity of these variations.

High frequency of BCG infection in our study also needs to be discussed. Yeganeh et al reported that complicated Bacillus Calmette-Guérin (BCG) vaccination was documented in 18 of 40 SCID cases (45%) following the routine vaccination at birth [29]. In another study, incidence of severe adverse reactions after BCG vaccination was reported as 0.0182 cases per 100,000 vaccinations in Japanese children. Among 39 cases with adverse reactions, 13 had been diagnosed to have some types of primary immunodeficiency. Four of them had SCID [30]. Sadeghi-Shanbestari M et al [31] have reported that seven of their eleven disseminated BCG infection and primary immunodeficiency patients were SCID cases. From these seven SCID and BCG infection cases, four patients had T-B-NK + SCID; probably RAG genes were involved [31]. The same authors have also reported a novel RAG2 mutation in a patient with SCID and BCG disease [32]. Four of our 11 RAGD cases (36.4%) had BCG disease (2 BCG-itis and 2 BCG-osis) and had to receive anti-mycobacterial therapy. The clinical findings and management of the patients who had documented BCG infection were worse than the rest of our group.

We reach to several conclusions from this review of our RAGD patients:

Epigenetic factors such as compound genetic defects, enviromental factors and exposure to infectious agents may modify phenotypical characteristics of RAGD. Homozygous mutation of *p.R394Q/p.R394Q and p.R776Q, 3047-3049 del GCC *mutations are novel and they are causing serious T-B-NK + SCID.

It is very important to increase the awareness of pediatricians for early diagnosis and treatment of these patients with RAG defects.

Inoculation of live vaccines such as BCG should be postponed in suspected cases for primary immunodeficiency disease, until appropriate screening tests exclude this diagnosis. Therefore, knowledge of previous cases with immunodeficiency within the same family is beneficial for early diagnosis and decision for postponing BCG in such patients.

## Competing interests

The authors declare that they have no competing interests.

## Authors' contributions

AB carried out the molecular genetic studies. NG and NEK reviewed the data and wrote the paper. NK and GA analyzed the data and edited the manuscript. All authors read and approved the final manuscript.
